# Development of an invasive ductal carcinoma in a contralateral composite nipple graft after an autologous breast reconstruction: a case report

**DOI:** 10.1186/s40792-020-00962-2

**Published:** 2020-08-08

**Authors:** Mariko Kimura, Kazutaka Narui, Hidetaka Shima, Shizune Ikejima, Mayu Muto, Toshihiko Satake, Mikiko Tanabe, Yoshiaki Inayama, Shoko Adachi, Akimitsu Yamada, Kazuhiro Shimada, Sadatoshi Sugae, Yasushi Ichikawa, Takashi Ishikawa, Itaru Endo

**Affiliations:** 1 Department of Breast and Thyroid Surgery, Yokohama City University Medical Center, 4-57 Urafune-cho, Minami-ku, Yokohama, 232-0024 Japan; 2 Plastic and Reconstructive Surgery, Yokohama City University Medical Center, Yokohama, Japan; 3 Diagnostic Pathology, Yokohama City University Medical Center, Yokohama, Japan; 4 Department of Gastroenterological Surgery, Yokohama City University Graduate School of Medicine, Yokohama, Japan; 5 Department of Oncology, Yokohama City University Graduate School of Medicine, Yokohama, Japan; 6 Department of Breast Oncology and Surgery, Tokyo Medical University, Shinjuku, Tokyo, Japan

**Keywords:** Breast cancer, Autologous breast reconstruction, Nipple graft, Contralateral breast cancer, Nipple-areola complex reconstruction, Deep inferior epigastric perforator flap, Reconstructed nipple, Donor nipple, Composite nipple grafting, Invasive cancer of graft

## Abstract

**Background:**

Nipple-areola complex (NAC) reconstruction is a technique used in breast reconstructive surgery, which is performed during the final stage of breast reconstruction after total mastectomy of primary breast cancer. Composite nipple grafts utilizing the contralateral NAC are common; however, to our knowledge, there are no reports of new primary invasive ductal carcinoma development within the graft. Here, we describe one such case for the first time.

**Case presentation:**

A 54-year-old woman was referred to us by the Department of Plastic and Reconstructive Surgery in our medical center for further evaluation of right nipple erosion. She had undergone total mastectomy of the right breast following a breast cancer diagnosis 15 years ago, at which time tumor biological profiling revealed the following: estrogen receptor (ER), positive; progesterone receptor (PgR), negative; and human epidermal growth factor receptor 2 (HER2), undetermined. She received adjuvant chemotherapy and endocrine therapy. She defaulted endocrine therapy for a few years, and 7 years after surgery, she underwent autologous breast reconstruction with a deep inferior epigastric perforator (DIEP) flap. In the following year, NAC reconstruction was performed using a composite graft technique. Seven years after the NAC reconstruction, erosion appeared on the nipple grafted from its contralateral counterpart; scrape cytology revealed malignancy. The skin on the right side of her chest around the NAC and subcutaneous fat tissue consisted of transferred tissue from the abdomen, as the DIEP flap and grafted nipple were located on the graft skin. The right nipple carcinoma arose from the tissue taken from the left nipple. Magnetic resonance imaging (MRI) or computed tomography showed no malignant findings in the left breast. As the malignant lesion seemed limited to the area around the grafted right nipple on MRI, surgical resection with sufficient lateral and deep margins was performed around the right nipple. Pathological findings revealed invasive ductal carcinoma with comedo ductal components infiltrating the graft skin and underlying adipose tissue. Immunohistochemistry revealed positive for ER, PgR, and HER2.

**Conclusions:**

To our knowledge, this is the first case involving the development of invasive ductal carcinoma in a nipple graft constructed on the skin of a DIEP flap, with the origin from the contralateral breast’s nipple.

## Background

Autologous breast reconstruction (ABR) after mastectomy for breast cancer has become common over the past decades [1]. An ABR with a perforator flap is a good option for a lot of patients who may be concerned about their body image, and nipple-areola complex (NAC) reconstruction is performed in women who have undergone a total or skin-sparing mastectomy, which differs from a nipple-sparing mastectomy. It is an integral part of the breast reconstruction process, as patients associate this stage with the end of their plastic surgery treatment and a sense of completion [2]. It was once a concern that surgical trauma could activate dormant micrometastases; however, more recent reports have shown no increased risk of breast cancer recurrence following breast reconstruction [3]. New primary carcinomas arising from the grafted breast tissue have rarely been reported following surgery for breast cancer. Here, we present a rare case of a new primary carcinoma in a reconstructed nipple, which originated from the contralateral nipple, without any malignancy detected through imaging in either the left nipple or left mammary gland.

## Case presentation

**Fig. 1:**
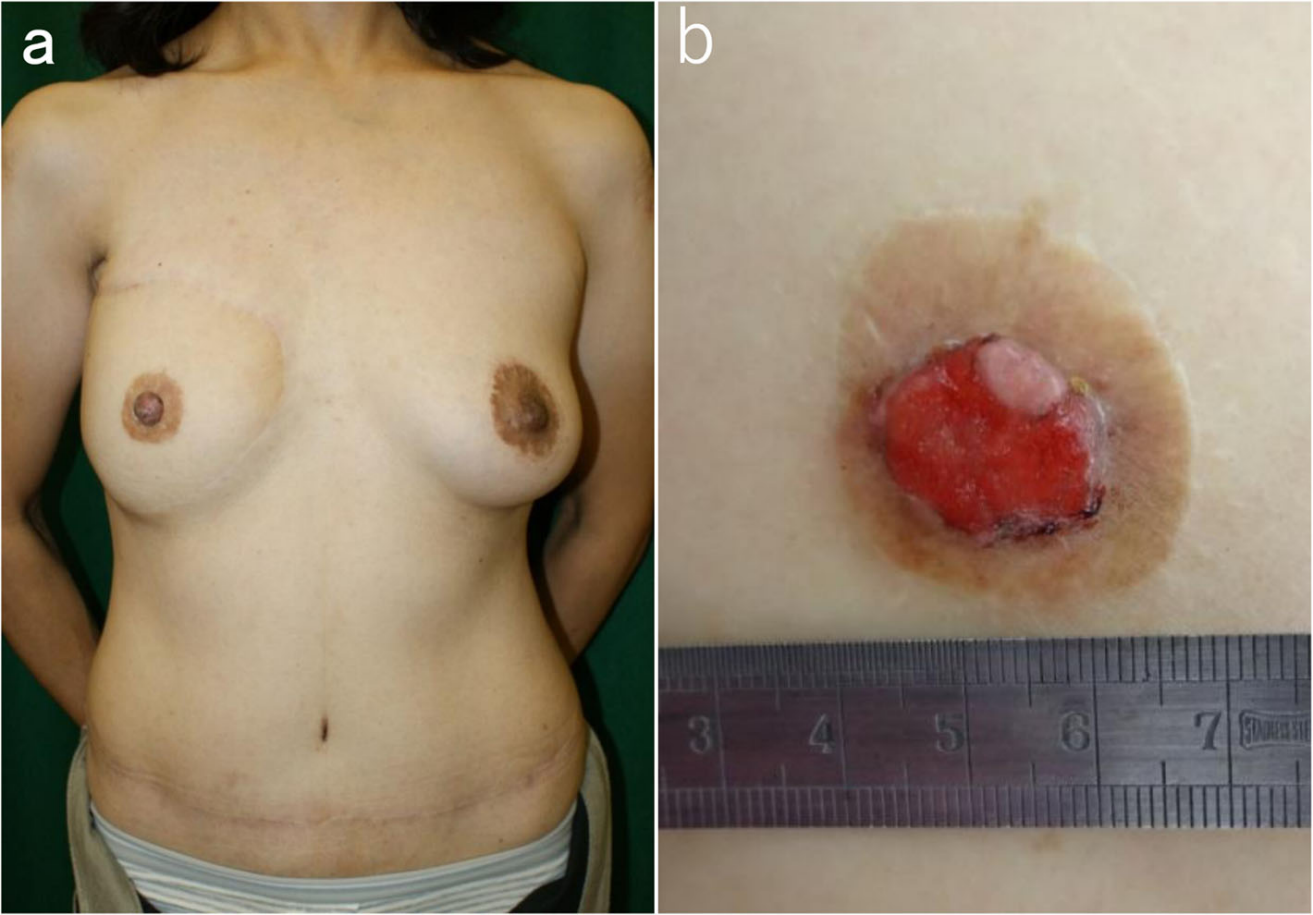
**a** This is an image obtained about 6 months after autologous breast reconstruction (ABR) with a deep inferior epigastric perforator (DIEP) flap, followed by nipple-areola complex (NAC) reconstruction of the right breast. **b** Image of the right NAC shows erosion and marginal crusting, with a small amount of normal tissue at the upper right side of the grafted nipple. The erosion was hemorrhagic on scraping. The grafted areola had no abnormality

A 54-year-old woman was referred to us by the Department of Plastic and Reconstructive Surgery in our medical center for further evaluation of prolonged right nipple erosion. She had previously received a diagnosis of right breast cancer and undergone total mastectomy and axillary dissection 15 years ago in another hospital. Histopathology identified an invasive ductal carcinoma with a tumor diameter of 0.9 cm and a nuclear grade of 2; one of 23 lymph nodes showed metastasis. Tissue profiling revealed the following: estrogen receptor (ER), positive; progesterone receptor (PgR), negative; and human epidermal growth factor receptor 2 (HER2), undetermined. As adjuvant therapy, she received six cycles of cyclophosphamide, methotrexate, and 5-fluorouracil (CMF), followed by tamoxifen for 3.5 years. Then, she defaulted her endocrine therapy. Seven years after the surgery, the doctors from our medical center performed ABR with a deep inferior epigastric perforator (DIEP) flap at another facility. The following year, her right nipple was reconstructed by V-shaped resection of the left nipple and an autologous grafting/nipple-sharing technique. The right areola was reconstructed with a penetrating skin graft from the proximal thigh and left areola. Concurrent mastopexy was performed for the left breast (Fig. 1a).

**Fig. 2:**
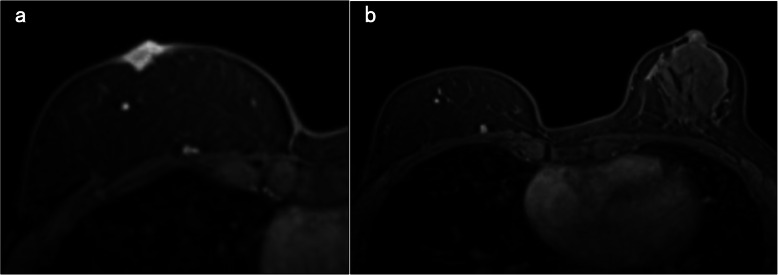
**a** Magnetic resonance imaging (MRI) shows enhancement of the lesion of the right nipple and slight subcutaneous adipose tissue. **b** There is no abnormal enhancement in the left breast

**Fig. 3:**
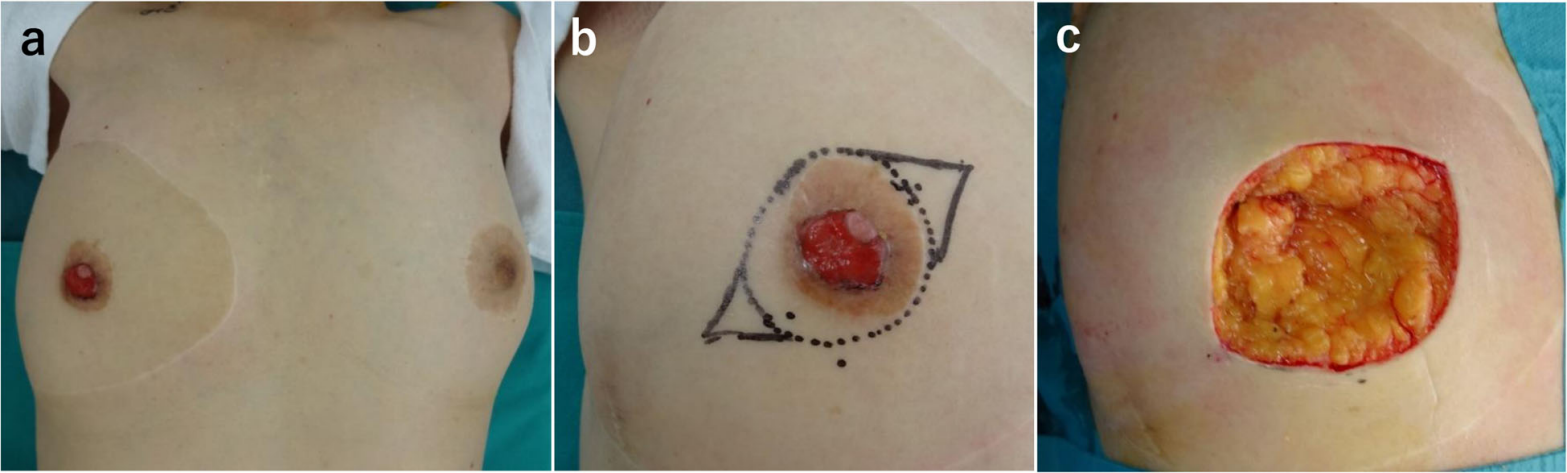
Preoperative image of the patient (**a**) and markings for skin incision (**b**). Intraoperative image after excision of the lesion with sufficient lateral and deep margins (**c**)

After 7.5 years, right nipple erosion appeared, and she visited the Department of Plastic and Reconstructive Surgery in our medical center. At first, it appeared that she had an eczematous nipple lesion caused by an infection, and she was treated with antibiotics; however, the erosion progressed and enlarged over the course of a few months. She was eventually referred to our department. The skin on the right side of her chest around the NAC and the subcutaneous adipose tissue consisted of transferred tissue from her abdomen, as the DIEP flap and grafted nipple were constructed on the skin graft. In the right nipple, normal tissue was almost completely affected by erosion, and there was no abnormality, itching, or pain in the right areola (Fig. 1b). Scrape cytology revealed malignancy of the epithelial cells, and that the right nipple carcinoma originated from the tissue taken from the left nipple. On magnetic resonance imaging (MRI), the malignant lesion seemed limited to the area around the grafted right nipple (Fig. 2a), with no malignancy observed in the left breast on MRI and computed tomography (CT) (Fig. 2b). In addition, no distant metastases were observed on CT. Paget’s disease was clinically suspected, and we performed surgical treatment. Though the standard surgical operation for mammary Paget’s disease is mastectomy, we performed partial breast excision including the right nipple with sufficient lateral and deep margins (Fig. 3a–c) because there was no mammary tissue in the right reconstructed breast, except for the nipple and areola. The incision was closed with investing sutures.

**Fig. 4:**
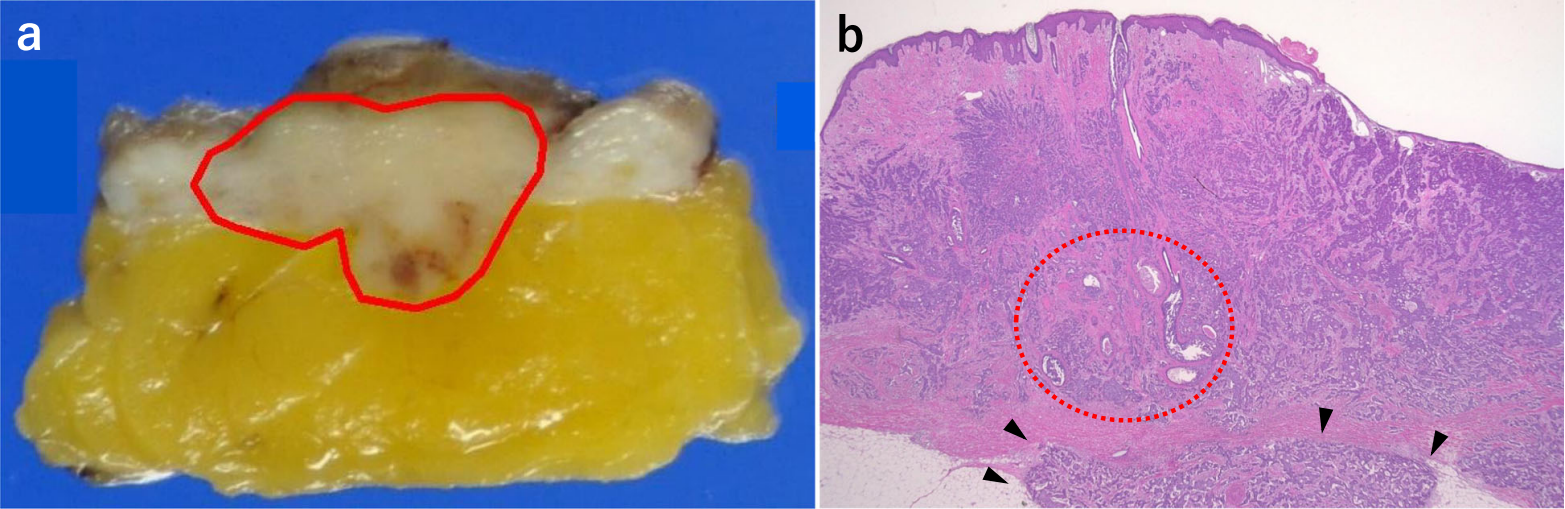
Histopathological findings. **a** The boundary of the tumor is delineated with a red line on the macroscopic image. The tumor is localized within epithelial and subcutaneous adipose tissue. The macroscopic image shows extensive infiltration and growth of tumor cells. **b** At the lower peripheral area, tumor cells are infiltrating into the adipose tissue across the desmoplastic border (black arrow). The infiltrating tumor cells show trabecular, sheet-like, acinar, and nesting growth patterns, with a few ductal carcinomas in situ with comedo necrosis (red dotted circle)

The specimen submitted for surgical pathology was composed of epithelial and adipose tissue grafted from the abdomen, areolar tissue grafted from the base of the thigh and left areola, and a nipple graft from the contralateral side. Macroscopically, the lesion spread around the nipple and adipose tissue (Fig. 4a). Pathological examination identified invasive ductal carcinoma with a few comedo ductal components within the nipple, extensive infiltration of grafted epithelial and adipose tissue (Fig. 4b), and a tumor diameter of 25 mm. The nuclear grade score was 2 (nuclear atypia score was 2 and mitotic count score was 2), there was no lymphatic or vascular invasion, and the lateral and deep margins were negative. Immunohistochemical staining showed strong positive for ER, weak positive for PgR, positive for HER2 with a score of 3+, and 35% cells showing positive Ki-67 staining.

As the biological profile classified the tumor as a luminal HER2 type, weekly paclitaxel, trastuzumab, and endocrine therapy were administered as adjuvant therapies. No distant metastases or local recurrence were seen 1 year after the surgery.

## Discussion

NAC reconstruction is a technique used in the final stage of breast reconstruction following total mastectomy for primary breast cancer. Composite nipple grafting has been described in the literature since the early 1970s, and the technique is now widely performed. Till date, only one report of breast cancer originating in a nipple reconstructed from tissue obtained from the contralateral/unaffected side has been identified via a PubMed search; however, that case involved Paget’s disease, not an invasive carcinoma [4]. A de novo carcinoma developed 6 years after the NAC reconstruction, and the donor nipple had no identifiable lesion, similar to the findings in this case. To our knowledge, that report documented the first case of Paget’s disease arising from a grafted nipple in the US literature.

Breast cancer is considered to develop from the epithelia of terminal duct lobular units (TDLUs), including cases of in situ and invasive carcinomas. TDLUs usually exist in distal mammary glands through a series of branches. On the other hand, major lactiferous ducts terminate in and exit from the mammary glands at the nipple, although there are cases where TDLUs exist in adjacent lactiferous ducts in the nipple. Kryvenko et al. investigated the frequency of TDLUs and occult neoplastic epithelial proliferation in grossly/clinically unremarkable nipples [5]. They observed TDLUs in 26% of nipples of tissue specimens from therapeutic or prophylactic mastectomies, while occult neoplastic epithelial proliferation was seen in 5% of grossly/clinically unremarkable, therapeutic mastectomy nipples. They also reported that the nipples were unremarkable in all cases of prophylactic mastectomies. Moreover, occult neoplastic proliferations in grossly unremarkable nipples were largely correlated with underlying carcinomas, but only two patients had a primary malignant neoplasm in their nipples. Based on these reports, it is difficult to confidently state whether grossly unremarkable donor nipples are afflicted by malignant neoplasms or not.

This case is also rare in terms of the origin of the invasive ductal carcinoma of the nipple. Very little has been published about breast cancers developing within the nipple, especially invasive ones. The first large series of carcinomas originating in the nipple was published in 1956 [6]. Of approximately 10,000 cases of malignant disease of the breast in patients undergoing surgery at the Mayo Clinic in 46 years, only 0.25% of cases originated in the nipple. Secondly, Sanders et al. have reported common features of invasive primary breast carcinomas originating in the nipple [7]. The frequency of those carcinomas was 0.3% in the report, and only 24 patients among more than 8000 breast carcinoma patients investigated in their study presented with symptoms related to the nipple. Of these patients, 42% had epithelial changes associated with in situ ductal carcinomas involving the skin of the nipple (Paget’s disease), with small foci of invasion into the dermis. The rest of the patients presented with a nipple mass with or without skin changes. Consistent with the case reported here, HER2 positivity was observed in tissue obtained from 60% of patients with Paget’s disease and 14% of patients with a nipple mass. The likelihood of lymph node metastases was not higher than that for carcinomas of similar sizes, and there was no disease recurrence following proper adjuvant therapy. Thus, invasive primary nipple carcinomas are rare, but conventional therapies for breast cancer treatment are also useful in such cases. In our case, we initially suspected Paget’s disease because of nipple erosion and the fact that no solid tumor was identified by palpation. The patient exhibited pagetoid spread of an invasive ductal carcinoma with comedo ductal components, without the presence of a palpable solid tumor. However, contrast scrape cytology suggested a ductal carcinoma because no melanin granules were observed in the malignant cells [8]. In contrast, considering that HER2 positivity is more frequent in Paget’s disease than in invasive ductal carcinoma, it cannot be denied that this carcinoma originated from Paget’s disease in terms of the immunoprofile. HER2 is overexpressed in 80–90%; ER and PgR are positive in approximately 40% and 30%, respectively, in Paget’s disease [9], in contrast with 15–30% positive for HER2 in invasive ductal carcinoma. However, there were only a few Paget cells within the squamous epithelium of the nipple, and most malignant cells had directly infiltrated the nipple surface, resulting in ulceration. In addition, the infiltrating malignant cells were not large cells with abundant eosinophilic cytoplasm like Paget cells. Therefore, they had likely originated from the invasive ductal carcinoma rather than Paget’s disease.

There has been a lot of discussion about the oncological safety of ABR after mastectomy for invasive breast cancer; however, a current literature review failed to show significant risks of either concurrent or delayed distant or local recurrence following ABR, relative to the risk in the absence of ABR. In this case, there were no remnant breast tissues in the right breast, except for the nipple and areola, and the malignant neoplasm arose from the flap of abdominal skin from a DIEP flap [10]. Thus, the malignancy was not defined as a local recurrence but as a new primary carcinoma, considering there were no right breast tissue components such as skin, blood, and lymphatic vessels around the malignant neoplasm. Although total mastectomy is usually recommended in cases of local recurrence of breast cancer, in this case, we performed a partial resection because the lesion was quite localized, and there was no mammary gland except for localized tissue beneath the nipple of the right breast, based on imaging, and the carcinoma seemed not to be related to lymphovascular invasion of the initial breast cancer.

It was unclear whether the de novo carcinoma arose before or after the NAC surgery. Surgical treatment might trigger the occurrence of malignant changes, although there are few case reports describing cancers developing in transferred tissues to support this scenario. Cancer development is a multi-faceted, dynamic series of events, and some new carcinogenic hypotheses have been described. Recently, the role of the microenvironment in driving tumor progression has been increasingly recognized [11]. Tumor formation begins when genetic abnormalities occur in cells that undergo rapid, unchecked proliferation. However, a tumor is not solely comprised of cancer cells; it is a heterogeneous collection of both cancer cells and surrounding noncancerous or stromal cells that work in concert with one another to promote unrestricted growth, infiltration, and propagation of malignancy throughout the body. In the microenvironment of carcinomas, tumor cells recruit various types of stromal cells such as fibroblasts, inflammatory cells, and endothelial cells, and their cellular interactions are important drivers of progressive tumor growth [12]. Expansion of the tumor stroma is often observed in invasive carcinomas; these changes result in desmoplasias, where tumors and stroma actively interact. Pathologically, our case presented scirrhous spread of a carcinoma in the absence of a palpable tumor; thus, we assumed that there was an expansion of a desmoplasia around those tissues.

A hypoxic environment is one of the key physiological and microenvironmental characteristics that differentiate tumors from normal tissues. The transcription factor hypoxia-inducible factor 1α and angiogenesis are important factors that regulate hypoxia-induced signaling cascades [13]. Hypoxia can drive and maintain genetic instability, resulting in a mutated phenotype. Moreover, hypoxia, along with acidosis, increases clonal selection, resulting in aggressive cancer phenotypes. As a result, stressors, including surgery, may induce oncogenic changes in tissue. In contrast, “nipple banking” was formally performed to rebuild a nipple, which is a technique in which a nipple is taken from a site on the ipsilateral breast, banked in the groin, and then later returned to the chest [14, 15]. Nevertheless, the nipple was taken in the case without apparent involvement of the nipple with carcinoma; some cases of the development of heterotopic carcinoma in the transplanted nipple were reported. This indicates that cancer cells in the transplanted nipple could survive in a hypoxic or ischemic environment. In our case, surgical stressors might also have affected the donor tissue of the left nipple, though the timing of generation of malignant cells was not definitive. Generally, careful preoperative screening for contralateral breast cancer should be performed before NAC reconstruction whether malignant lesions exist, though there were no signs of malignancy in the left breast in this case. Moreover, post-operative examinations are important because of the possibility of development of occult malignant tumors in the left breast; thus, we have continued to monitor the patient’s left breast carefully.

## Conclusions

To our knowledge, this is the first report of an invasive ductal carcinoma developing in a grafted nipple following ABR. This might be a rare case, but clinicians must consider the possibility that carcinomas can develop in a graft as long as there are remnants of mammary gland tissue within the graft.

## Abbreviations

ABR: Autologous breast reconstruction
CMF: Cyclophosphamide, methotrexate, and 5-fluorouracil (5-FU)
CT: Computed tomography
DIEP: Deep inferior epigastric perforator
ER: Estrogen receptor
HER2: Human epidermal growth factor receptor 2
MRI: Magnetic resonance imaging
NAC: Nipple-areola complex
PgR: Progesterone receptor
TDLU: Terminal duct lobular unit
